# Molecular identification of tobacco leaf curl disease in Sichuan province of China

**DOI:** 10.1186/s12985-015-0461-7

**Published:** 2016-01-06

**Authors:** Chenchen Jing, Chunyan Wang, Ke Li, Gentu Wu, Xianchao Sun, Ling Qing

**Affiliations:** Chongqing Key Laboratory of Plant Disease Biology, College of Plant Protection, Southwest University, Chongqing, 400716 People’s Republic of China

**Keywords:** Tobacco, Tomato yellow leaf curl China virus, Papaya leaf curl China virus, Co-infection

## Abstract

**Background:**

Tobacco leaf curl disease (TLCD) is caused by begomoviruses in *Geminiviridae*, and infected plants exhibit leaf thickening, downward leaf curling, vein swelling as well as stunting symptoms. It is one of the economically important diseases in tropical and subtropical tobacco-growing areas. Seven monopartite begomoviruses have been identified causing TLCD in China.

**Findings:**

In this study, two begomoviruses were identified, characterized and polygenetically analyzed to be responsible for TLCD in Sichuan province, China. The complete genomes of two isolates SC230 and SC379 from diseased tobacco samples were cloned and sequenced to be 2738 nucleotides (nts) and 2748 nts in size, respectively. Sequence alignment indicated that SC230 and SC379 were most closely related to Tomato yellow leaf curl China virus (TYLCCNV-CN[CN:Sc226:Mal:12]) and Papaya leaf curl China virus (PaLCuCNV-CN[CN:Gx30:Lyc:03]), with a sequence identity of 99.2 and 99.2 %, respectively. The infection rate of TYLCCNV and PaLCuCNV was 100 and 34.78 %, respectively and the co-infection rate was 34.78 % in fields. Betasatellites of SC230 and SC379 share the highest sequence identity with Tomato yellow leaf curl China betasatellite (TYLCCNB-CN[CN:Sc176:Malva:12]) and TYLCCNB-CN[CN:Yn149:Tom:09], with a sequence identity of 95.2 and 97.2 % respectively. Sequence identity between betasatellites of SC230 and SC379 was 89.6 %. And TYLCCNB was detected in all the samples.

**Conclusion:**

Co-infection of TYLCCNV and PaLCuCNV was identified in tobacco plants with typical symptoms of TLCD from Sichuan province in China, and this is the first report of PaLCuCNV infecting tobacco in China. TYLCCNV/TYLCCNB disease complex is widespread in tobacco-growing areas in Panzhihua city of Sichuan.

**Electronic supplementary material:**

The online version of this article (doi:10.1186/s12985-015-0461-7) contains supplementary material, which is available to authorized users.

## Background

Geminiviruses are a large family of plant viruses with circular, single-stranded DNA genome encapsidated in unique twinned particles. They consist of seven genera (*Mastrevirus*, *Curtovirus*, *Topocuvirus*, *Begomovirus*, *Becurtovirus*, *Eragrovirus* and *Turncurtovirus*) on the basis of genome structure, host range and insect vector [1, 2]. Most of the economically important geminiviruses belong to the genus *Begomovirus*, which are transmitted by *Bemisia tabaci* to a wide range of dicotyledonous plants [3]. Begomoviruses cause significant economic losses to many crops, including common bean, cotton, cucurbits, okra, tomato, pepper and tobacco [4–7]. In China, TLCD caused by begomoviruses was first observed in Yunnan and Fujian province in 1982 [8]. Recently, TLCD has occurred in Guangxi and Guangdong [9, 10]. So far seven distinct monopartite begomoviruses have been identified causing TLCD in China, including TYLCCNV, Tobacco leaf curl virus (TbLCV), Tomato leaf curl China virus (ToLCCNV), Tobacco leaf curl Yunnan virus (TLCYNV), Tobacco curly shoot virus (TbCSV), Ageratum yellow vein virus (AYVV) and Papaya leaf curl Guangdong virus (PaLCuGuV) [9–15].

Recently, the occurrence of begomoviruses in Sichuan is more and more frequent. TbCSV was firstly reported infecting pepper. The co-infection of TYLCCNV/TYLCCNB and PaLCuCNV was identified in tomato, which caused serious tomato yellow leaf curl disease (TYLCD), and TYLCCNV/TYLCCNB was found infecting *Malva rotundifolia* Linn. *Malvastrum coromandelianum*, a widespread weed in tropical regions, was infected by TYLCCNV, Malvastrum yellow vein Yunnan virus (MYVYNV) and Malvastrum yellow vein virus (MYVV) associated betasatellite [7, 16–18]. However none of begomoviruses was found on tobacco. In this study we report the occurrence of TLCD associated begomoviruses in Panzhihua city of Sichuan province, and the infection of PaLCuCNV in tobacco for the first time in China.

## Methods

**Fig. 1:**
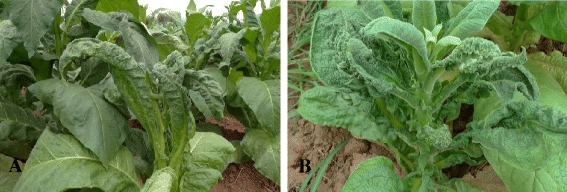
The typical symptoms of tobacco plant infected by tobacco leaf curl disease in the field. **a** The symptoms of tobacco plant infected by TYLCCNV + TYLCCNB; **b** The symptoms of tobacco plant infected by PaLCuCNV + TYLCCNV + TYLCCNB

Twenty three samples of tobacco plants with typical leaf thickening, downward leaf curling, vein swelling, yellow vein and stunting symptoms were collected in two fields at a distance of about 80 kilometers in Panzhihua city of Sichuan province (southwestern China) in August, 2012, including eight samples showing very severe leaf thickening, leaf crinkling, enation and stunting symptoms (Fig. 1) [see Additional file 1]. In addition, four symptomless tobacco plants were randomly collected from the same fields.

Total DNA was extracted using CTAB method. Degenerate primer pair PA/PB specific for members of the genus *Begomovirus* was used for detection [19]. The PCR products were cloned into pGEM-T Easy Vector (Promega, Madison, WI, USA) and four clones were randomly selected and sequenced. The sequencing of clones was performed by DNA sequencer in Beijing Genomics Institute. Sequences were assembled and analyzed with the aid of the DNAStar software version 6.0 (DNAStar Inc., Madison, WI, USA) and MEGA 6.0.6.

To identify the possible viruses in these tobacco samples, the specific primer pairs of begomoviruses which reported occurring in Sichuan province were either designed based on their conserved region obtained from multiple sequence alignment of the corresponding isolates in GenBank or cited from previous studies. These primer pairs are specific for PaLCuCNV (YM1P-F: 5′-TCACAAACAAAAGGAGGTCA-3′/YM1P-R: 5′-GAATATGTAACACATTCAA-3′), MYVV (MY-F: 5′-GCCCACTAACTACTGTCTG-3′/MY-R: 5′-TTAGAGGCATGGGTACATGC-3′), MYVYNV (MYV-F: 5′-GTTCTTTGGTTGAGGCAG-3′/MYV-R: 5′-TTAGAGGCATGGGTACATGC-3′), TbCSV (TbCSV-F/YnR) and TYLCCNV (TYLCCNV-F/YnR) [20], respectively.

Overlapping primers SC379-Pa-F (5′-GTTGTATATGGCATGTACTCATGC-3′) SC379-Pa-R (5′-CCGATACATCCTGGGCTTTCGGTA-3′) and TYLCCNV-F (5′-ACCGGATGTACAGAAGCCCTGA-3′)/TYLCCNV-R (5′-ATGTACCGGAAGCCCATGATGTAC-3′) were designed for amplification of full length DNA of PaLCuCNV and TYLCCNV, respectively. The universal abutting primers beta01 and beta02 were used to amplify the DNA molecules of betasatellites [21]. The primer pairs Y10beta (5′-CGGCATTATTTTGAGGCAGT-3′)/beta02 were used for specific amplification of Tomato yellow leaf curl China betasatellite (TYLCCNB). Products of PCR amplification of viral DNA and betasatellite were cloned and sequenced. All of the sequences used for comparison were obtained from GenBank. The sequences of begomoviruses and betasatellites, occurred in Sichuan and causing TLCD in China, were selected for construction of phylogenetic tree [see Additional file 2 & Additional file 3]. Phylogenetic trees were constructed by the neighbour-joining method with 1000 bootstrap replications using MEGA6.0.6.

## Results and discussion

A 500 bp DNA fragment covering parts of the intergenic region (IR) and *V2* gene of the genomes of begomoviruses was amplified from all 23 symptomatic samples using degenerate primer pair PA/PB. No positive fragment was detectable in any symptomless samples. Four clones from isolate SC225, SC230, SC378, SC240 were randomly selected to be sequenced [GenBank: KF640690, KF640691, KF640692 and KF640693]. The sequence comparison revealed that the 4 clones were most closely related to TYLCCNV, with a sequence identity ranges from 94.5 to 97.7 %.

In order to identify the occurrence of the other previously reported begomoviruses in Sichuan in symptomatic samples, PCR detection with specific primer pairs was conducted and results showed that only TYLCCNV (in all 23 samples) and PaLCuCNV (in 8 samples of SC228, SC230, SC232, SC236, SC238, SC240, SC242 and SC379) were detectable, and the co-infection rate of PaLCuCNV and TYLCCNV was 34.78 % [see Additional file 1].

**Fig. 2:**
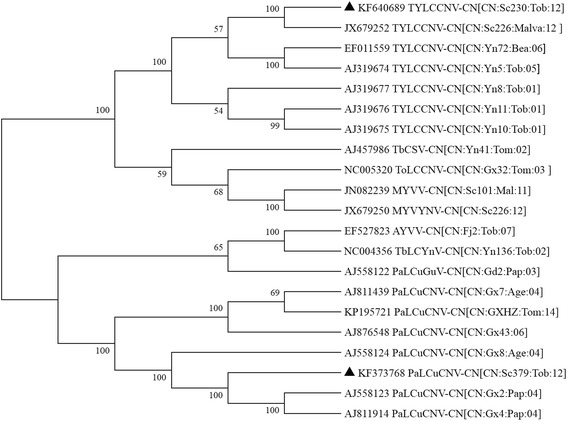
Phylogenetic tree of complete DNA of TYLCCNV-CN[CN:Sc230:Tob:12] and PaLCuCNV-CN[CN:Sc379:Tob:12]. The phylogenetic tree was constructed with neighbour-joining method with 1000 bootstrap replications and viewed with the help of MEGA 6.0.6. Triangle symbol indicates the position of TYLCCNV-CN[CN:Sc230:Tob:12] and PaLCuCNV-CN[CN:Sc379:Tob:12]

The complete DNA sequences of isolate SC230 and SC379 were determined to be 2738 nts [GenBank: KF640689] and 2748 nts [GenBank: KF373768], respectively. A comparison with other begomoviruses showed that DNA of SC230 was closely related to TYLCCNV-CN [CN:Sc226:Mal:12] (99.2 % identity), and SC379 was closely related to PaLCuCNV-CN[CN:Gx30:Lyc:03] (99.2 % identity). The genomic organization of two DNA sequences were typical of the Old World begomoviruses, with two ORFs (*AV2* and *AV1*) in the virion-sense strand and four ORFs (*AC1* to *AC4*) in the complementary-sense strand, which separated by the intergenic region (IR). The IR contains a putative stem-loop structure sequence with the conserved nonanucleotide sequence TAATATTAC in the loop, and this motif contains the nicking site for the initiation of rolling circle replication [22]. Generally, the isolates of begomoviruses showing more than 91 % identity are considered to be strains of the same species [23]. SC230 and SC379 are thus considered to be isolates of TYLCCNV and PaLCuCNV respectively, and we suggested the name that TYLCCNV-CN[CN:Sc230:Tob:14] and PaLCuCNV-CN[CN:C379:Tob:12]. The relationship dendrogram of the complete nucleotide sequences of PaLCuCNV-CN[CN:C379:Tob:12], TYLCCNV-CN[CN:Sc230:Tob:14] and other begomoviruses indicates that PaLCuCNV-CN[CN:C379:Tob:12] was grouped into a single cluster together with PaLCuCNV-CN[CN:Gx2:Pap:04] and PaLCuCNV-CN[CN:Gx4:Pap:04], and TYLCCNV-CN[CN:Sc230:Tob:14] was grouped into a single cluster together with TYLCCNV-CN[CN:Sc226:Mal:12] (Fig. 2).

**Fig. 3:**
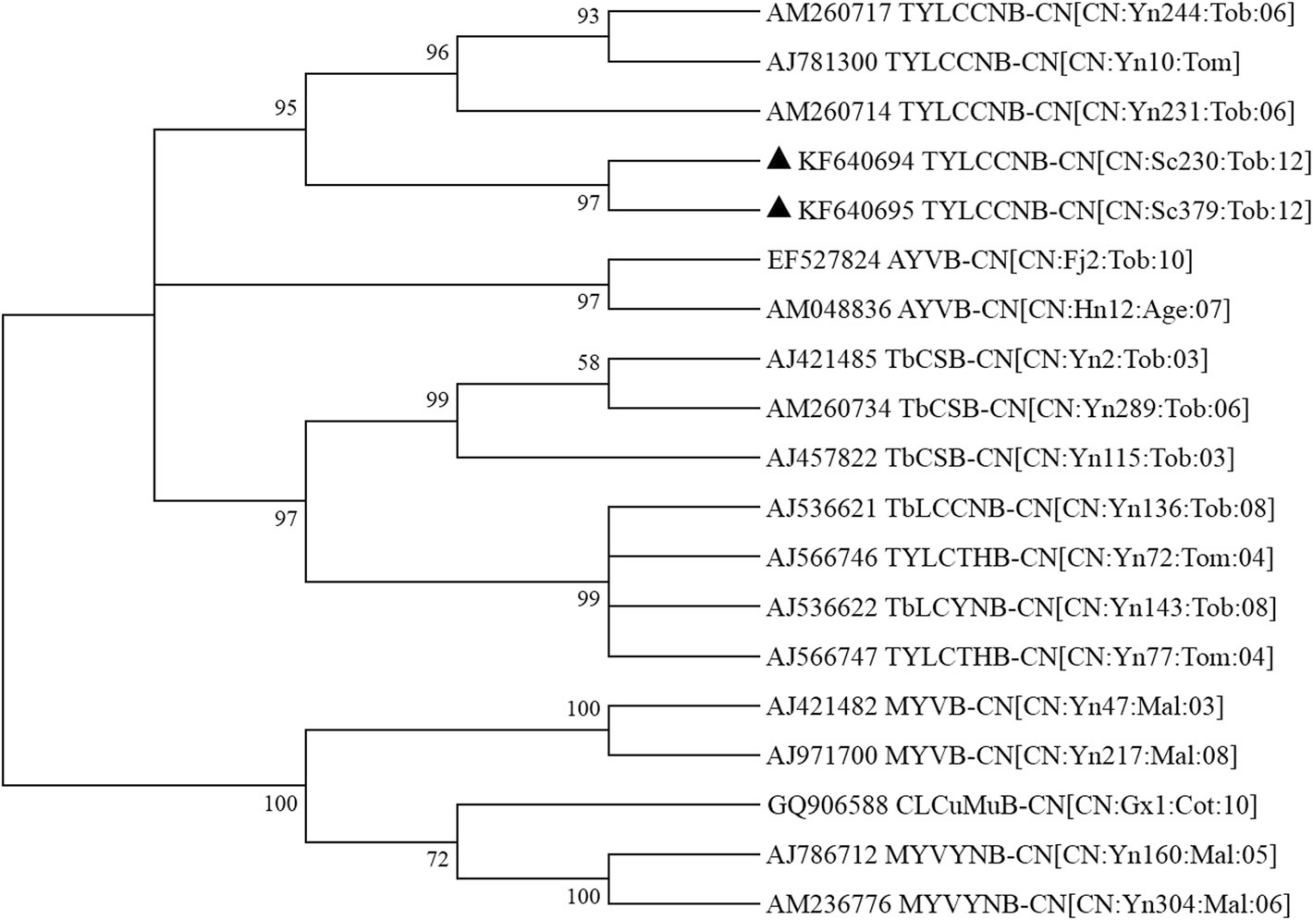
Phylogenetic tree of betasatellites associated with TYLCCNV-CN[CN:Sc230:Tob:12] and PaLCuCNV-CN[CN:Sc379:Tob:12]. The phylogenetic tree was constructed with neighbour-joining method with 1000 bootstrap replications and viewed with the help of MEGA 6.0.6. Triangle symbol indicates the positions of TYLCCNB-CN[CN:Sc379:Tob:12] and TYLCCNB-CN[CN:Sc230:Tob:12]

With the primers beta01 and beta02 for betasatellite DNA, an amplicon of 1300 bp was obtained from all symptomatic samples and none of fragment was detectable in symptomless samples. Sequence comparison showed that the betasatellite from SC379 is 1339 bp long [GenBank: KF640695] and had the highest sequence identity with Tomato yellow leaf curl China betasatellite (TYLCCNB-CN[CN:Yn149:09]) 97.2 % identity), and the betasatellite from SC230 is 1337 bp long [GenBank: KF640694] and had the highest sequence identity with TYLCCNB-CN[CN:Sc176:Malva:12] (95.2 % identity). These results suggested that both of betasatellites are isolates of TYLCCNB. The sequence identity between TYLCCNB-CN[CN:Sc379:12] and TYLCCNB-CN[CN:Sc230:12] was 89.6 %. These two molecules of betasatellite were consistently clustered with the TYLCCNB isolates (Fig. 3). And TYLCCNB was detectable in all symptomatic samples by PCR using specific primer pair Y10beta/beta02.

In general, co-infection of TYLCCNV and PaLCuCNV were detected in tobacco plants with typical symptoms of TLCD, and TYLCCNV/TYLCCNB disease complexes are widespread in infected tobacco plants in Panzhihua city of Sichuan province. These results were consistent with other reports in which PaLCuCNV is a monopartite begomovirus with no satellite, and TYLCCNV is always associated with its cognate satellite TYLCCNB forming begomovirus/betasatellite disease complex [24, 25]. TLCD commonly occurs in Yunnan, Fujian, Guangxi and Guangdong provinces in China and has a trend of spreading. In Sichuan province, TYLCD has caused great losses of tomato cultivation, but TLCD has not been found on tobacco so far. To our knowledge, PaLCuCNV commonly infects tomato, *Ageratum conyzoides*, *Corchoropsis tomentosa* and papaya, TYLCCNV/TYLCCNB complex infects tomato, tobacco, *Datura stramonium L.*, *Siegesbeckia orientalis*, *Solanum aculeatissimum* and *Malva rotundifolia* Linn. in China [17, 26–33]. In this study, we reported occurrence of TLCD in Sichuan province and PaLCuCNV infecting tobacco for the first time in China, and identified co-infection of PaLCuCNV and TYLCCNV associated with TYLCCNB. Previous studies have revealed that betasatellite could be *trans*-replicated by noncognate begomoviruses and the host range of begomoviruses could be enlarged while associated with betasatellite [34, 35]. In this study, co-infection of TYLCCNV and PaLCuCNV was only detected in the 8 samples with more severe leaf thickening, leaf crinkling, enation and stunting symptoms, which is consistant with the previous report that the tomato yellow leaf curl disease caused by co-infection of TYLCCNV and PaLCuCNV is more severe than that caused by TYLCCNV/TYLCCNB. The results suggested the synergism between TYLCCNV and PaLCuCNV probably occurred. Therefore, it would be interesting to illustrate the role of TYLCCNB on the infection of PaLCuCNV to the new host and the interaction between TYLCCNV and PaLCuCNV in the future.

## Additional files

Additional file 1:
**The typical symptoms and polymerase chain reaction detection of five begomoviruses and betasatellite in tobacco samples.** (DOCX 16 kb) Additional file 2:
**Details of DNA sequences of begomoviruses selected from GenBank for phylogenetic analysis in this study.** (DOCX 17 kb) Additional file 3:
**Details of betasatellite sequences of begomoviruses selected from GenBank for phylogenetic analysis in this study.** (DOCX 16 kb)
