# Supplementary material for: Molecular identification of tobacco leaf curl disease in Sichuan province of China
Source: Virol J. 2016 Jan 6;13:4. doi: 10.1186/s12985-015-0461-7 (PMC4704257; doi:10.1186/s12985-015-0461-7)
Supplement: Additional file 1: — The typical symptoms and polymerase chain reaction detection of five begomoviruses and betasatellite in tobacco samples. (DOCX 16 kb) [file 12985_2015_461_MOESM1_ESM.docx]

**Additional file 1 The typical symptoms of tobacco plants and polymerase chain reaction detection of five begomoviruses and betasatellite in tobacco samples**

| **Samples** | **Symptoms** | **Detection of viral or betasatellite DNA** | | | | | |
| --- | --- | --- | --- | --- | --- | --- | --- |
|  |  | **PaLCuCNV** | **MYVV** | **MYVYNV** | **TbCSV** | **TYLCCNV** | **TYLCCNB** |
| SC225 | leaf thickening, downward leaf curling, yellow vein, vein swelling, vein darkening, enation and stunting | - | - | - | - | + | + |
| SC226 | leaf thickening, downward leaf curling, yellow vein, vein swelling, vein darkening, enation and stunting | - | - | - | - | + | + |
| SC227 | leaf thickening, downward leaf curling, yellow vein, vein swelling, vein darkening, enation and stunting | - | - | - | - | + | + |
| SC228 | leaf thickening, yellow vein, vein swelling, vein darkening, enation and severe stunting, downward leaf curling and leaf crinkling | + | - | - | - | + | + |
| SC229 | leaf thickening, downward leaf curling, yellow vein, vein swelling, vein darkening, enation and stunting | - | - | - | - | + | + |
| SC230 | leaf thickening, yellow vein, vein swelling, vein darkening, enation and severe stunting, downward leaf curling and leaf crinkling | + | - | - | - | + | + |
| SC231 | leaf thickening, downward leaf curling, yellow vein, vein swelling, vein darkening, enation and stunting | - | - | - | - | + | + |
| SC232 | leaf thickening, yellow vein, vein swelling, vein darkening, enation, severe stunting and downward leaf curling | + | - | - | - | + | + |
| SC233 | leaf thickening, downward leaf curling, yellow vein, vein swelling, vein darkening, enation and stunting | - | - | - | - | + | + |
| SC234 | leaf thickening, downward leaf curling, yellow vein, vein swelling, vein darkening, enation and stunting | - | - | - | - | + | + |
| SC235 | leaf thickening, downward leaf curling, yellow vein, vein swelling, vein darkening, enation and stunting | - | - | - | - | + | + |
| SC236 | leaf thickening, yellow vein, vein swelling, vein darkening, enation and severe stunting, downward leaf curling and leaf crinkling | + | - | - | - | + | + |
| SC237 | leaf thickening, downward leaf curling, yellow vein, vein swelling, vein darkening, enation and stunting | - | - | - | - | + | + |
| SC238 | leaf thickening, yellow vein, vein swelling, vein darkening, enation, severe stunting and downward leaf curling | + | - | - | - | + | + |
| SC239 | leaf thickening, downward leaf curling, yellow vein, vein swelling, vein darkening, enation and stunting | - | - | - | - | + | + |
| SC240 | leaf thickening, yellow vein, vein swelling, vein darkening, enation and severe stunting, downward leaf curling and leaf crinkling | + | - | - | - | + | + |
| SC241 | leaf thickening, downward leaf curling, yellow vein, vein swelling, vein darkening, enation and stunting | - | - | - | - | + | + |
| SC242 | leaf thickening, yellow vein, vein swelling, vein darkening, enation and severe stunting, downward leaf curling and leaf crinkling | + | - | - | - | + | + |
| SC243 | leaf thickening, downward leaf curling, yellow vein, vein swelling, vein darkening, enation and stunting | - | - | - | - | + | + |
| SC377 | leaf thickening, downward leaf curling, yellow vein, vein swelling, vein darkening, enation and stunting | - | - | - | - | + | + |
| SC378 | leaf thickening, downward leaf curling, yellow vein, vein swelling, vein darkening, enation and stunting | - | - | - | - | + | + |
| SC379 | leaf thickening, yellow vein, vein swelling, vein darkening, enation and severe stunting, downward leaf curling and leaf crinkling | + | - | - | - | + | + |
| SC380 | leaf thickening, downward leaf curling, yellow vein, vein swelling, vein darkening, enation and stunting | - | - | - | - | + | + |
| SC244 | Regular growth | - | - | - | - | - | - |
| SC245 | Regular growth | - | - | - | - | - | - |
| SC381 | Regular growth | - | - | - | - | - | - |
| SC382 | Regular growth | - | - | - | - | - | - |

+, viral DNA was obtained; –, no viral DNA was obtained.
