# Supplementary material for: Molecular identification of tobacco leaf curl disease in Sichuan province of China
Source: Virol J. 2016 Jan 6;13:4. doi: 10.1186/s12985-015-0461-7 (PMC4704257; doi:10.1186/s12985-015-0461-7)
Supplement: Additional file 2: — Details of DNA sequences of begomoviruses selected from GenBank for phylogenetic analysis in this study. (DOCX 17 kb) [file 12985_2015_461_MOESM2_ESM.docx]

**Additional file 2 Details of DNA sequences of begomoviruses selected from GenBank for phylogenetic analysis in this study**

| **Begomoviruses** | **GenBank accession #** | **Acronym** |
| --- | --- | --- |
| Tomato yellow leaf curl China virus-China[China:Sichuan230:Tobacco:2012] | KF640689 | TYLCCNV-CN[CN:Sc230:Tob:12] |
| Papaya leaf curl China virus-China[China: Sichuan379:Tobacco:2012] | KF373768 | PaLCuCNV-CN[CN:C379:Tob:12] |
| Tomato yellow leaf curl China virus-China[China:Sichuan226: Malva:2012] | JX679252 | TYLCCNV-CN[CN:Sc226:Malva:12 ] |
| Tomato yellow leaf curl China virus-China[China:Yunnan72:Bean:2006] | EF011559 | TYLCCNV-CN[CN:Yn72:Bea:06] |
| Tomato yellow leaf curl China virus-China[China:Yunnan5:Tobacco:2005] | AJ319674 | TYLCCNV-CN[CN:Yn5:Tob:05] |
| Tomato yellow leaf curl China virus-China[China:Yunnan8:Tobacco:2001] | AJ319677 | TYLCCNV-CN[CN:Yn8:Tob:01] |
| Tomato yellow leaf curl China virus-China[China:Yunnan11:Tobacco:2001] | AJ319676 | TYLCCNV-CN[CN:Yn11:Tob:01] |
| Tomato yellow leaf curl China virus-China[China:Yunnan10:Tobacco:2001] | AJ319675 | TYLCCNV-CN[CN:Yn10:Tob:01] |
| Tobacco curl shoot virus-China[China:Yunnan41:Tomato:2002] | AJ457986 | TbCSV-CN[CN:Yn41:Tom:02] |
| Tomato leaf curl China virus-China[China:Guangxi32: Tomato:2001] | NC005320 | ToLCCNV-CN[CN:Gx32:Tom:03 ] |
| Malvastrum yellow vein virus-China[China: Sichuan101: Malvastrum:2011] | JN082239 | MYVV-CN[CN:Sc101:Mal:11] |
| Malvastrum yellow vein Yunnan virus-China[China: Sichuan226:2011] | JX679250 | MYVYNV-CN[CN:Sc226:12] |
| Ageratum yellow vein virus-China[China:Fujian2:Tobacco:2007] | EF527823 | AYVV-CN[CN:Fj2:Tob:07] |
| Tobacco curl Yunnan virus-China[China:Yunnan136:Tobacco:2002] | NC004356 | TbLCYnV-CN[CN:Yn136:Tob:02] |
| Papaya leaf curl Guangdong virus-China[China:Guangdong2:Papaya:2003] | AJ558122 | PaLCuGuV-CN[CN:Gd2:Pap:03] |
| Papaya leaf curl China virus-China[China:Guangxi7:Ageratum:2004] | AJ811439 | PaLCuCNV-CN[CN:Gx7:Age:04] |
| Papaya leaf curl China virus-China[China:GXHZ:Papaya:2014] | KP195721 | PaLCuCNV-CN[CN:GXHZ:Tom:14] |
| Papaya leaf curl China virus-China[China:Gx43: Corchoropsis:2006] | AJ876548 | PaLCuCNV-CN[CN:Gx43:Cor:06] |
| Papaya leaf curl China virus -China[China:Guangxi8:Ageratum:2004] | AJ558124 | PaLCuCNV-CN[CN:Gx8:Age:04] |
| Papaya leaf curl China virus -China[China:Guangxi2:Papaya:2004] | AJ558123 | PaLCuCNV-CN[CN:Gx2:Pap:04] |
| Papaya leaf curl China virus -China[China:Guangxi4:Papaya:2004] | AJ811914 | PaLCuCNV-CN[CN:Gx4:Pap:04] |
| Papaya leaf curl China virus -China[China:Guangxi30:Tomato:2003] | AJ558117 | PaLCuCNV-CN[CN:Gx30:Tom:03] |
