# Supplementary material for: Molecular identification of tobacco leaf curl disease in Sichuan province of China
Source: Virol J. 2016 Jan 6;13:4. doi: 10.1186/s12985-015-0461-7 (PMC4704257; doi:10.1186/s12985-015-0461-7)
Supplement: Additional file 3: — Details of betasatellite sequences of begomoviruses selected from GenBank for phylogenetic analysis in this study. (DOCX 16 kb) [file 12985_2015_461_MOESM3_ESM.docx]

**Additional file 3 Details of betasatellite sequences of begomoviruses selected from GenBank for phylogenetic analysis in this study**

| **Betasatellites** | **GenBank accession #** | **Acronym** |
| --- | --- | --- |
| Tomato leaf curl China betasatellite-China[China:Sichuan230:Tobacco:2012] | KF640694 | TYLCCNB-CN[CN:Sc230:Tob:12] |
| Tomato leaf curl China betasatellite-China[China:Sichuan379:Tobacco:2012] | KF640695 | TYLCCNB-CN[CN:Sc379:Tob:12] |
| Ageratum yellow vein betasatellite-China[China:Fujian2:Tobacco:2010] | EF527824 | AYVB-CN[CN:Fj2:Tob:10] |
| Ageratum yellow vein betasatellite-China[China:Henan12:Ageratum:2007] | AM048836 | AYVB-CN[CN:Hn12:Age:07] |
| Cotton leaf curl Multan betasatellite-China[China:Guangxi1:Cotton:2010] | GQ906588 | CLCuMuB-CN[CN:Gx1:Cot:10] |
| Malvastrum yellow vein betasatellite-China[China:Yunnan47:Malvastrum:2003] | AJ421482 | MYVB-CN[CN:Yn47:Mal:03] |
| Malvastrum yellow vein betasatellite-China[China:Yunnan217:Malvastrum:2008] | AJ971700 | MYVB-CN[CN:Yn217:Mal:08] |
| [Malvastrum yellow vein Yunnan](http://www.ncbi.nlm.nih.gov/Taxonomy/Browser/wwwtax.cgi?id=377611) betasatellite-China[China:Yunnan160:Malvastrum:2005] | AJ786712 | MYVYNB-CN[CN:Yn160:Mal:05] |
| [Malvastrum yellow vein Yunnan](http://www.ncbi.nlm.nih.gov/Taxonomy/Browser/wwwtax.cgi?id=377611) betasatellite-China[China:Yunnan304:Malvastrum:2006] | AM236776 | MYVYNB-CN[CN:Yn304:Mal:06] |
| [Tobacco curly shoot betasatellite](http://www.ncbi.nlm.nih.gov/Taxonomy/Browser/wwwtax.cgi?id=219596)-China[China:Yunnan2:Tobacco:2003] | AJ421485 | TbCSB-CN[CN:Yn2:Tob:03] |
| [Tobacco curly shoot betasatellite](http://www.ncbi.nlm.nih.gov/Taxonomy/Browser/wwwtax.cgi?id=219596)-China[China:Yunnan115:Tobacco:2003] | AJ457822 | TbCSB-CN[CN:Yn115:Tob:03] |
| [Tobacco curly shoot betasatellite](http://www.ncbi.nlm.nih.gov/Taxonomy/Browser/wwwtax.cgi?id=219596)-China[China:Yunnan289:Tobacco:2006] | AM260734 | TbCSB-CN[CN:Yn289:Tob:06] |
| Tobacco leaf curl China betasatellite -China[China:Yunnan136:Tobacco:2008] | AJ536621 | TbLCCNB-CN[CN:Yn136:Tob:08] |
| Tobacco leaf curl Yunnan betasatellite-China[China:Yunnan143:Tobacco:2008] | AJ536622 | TbLCYNB-CN[CN:Yn143:Tob:08] |
| Tomato yellow leaf curl Thailand betasatellite-China[China:Yunnan72:Tomato:2004] | AJ566746 | TYLCTHB-CN[CN:Yn72:Tom:04] |
| Tomato yellow leaf curl Thailand betasatellite-China[China:Yunnan77:Tomato:2004] | AJ566747 | TYLCTHB-CN[CN:Yn77:Tom:04] |
| Tomato leaf curl China betasatellite-China[China:Yunnan244:Tobacco:2006] | AM260717 | TYLCCNB-CN[CN:Yn244:Tob:06] |
| Tomato leaf curl China betasatellite-China[China:Yunnan231:Tobacco:2006] | AM260714 | TYLCCNB-CN[CN:Yn231:Tob:06] |
| Tomato leaf curl China betasatellite-China[China:Yunnan:Tomato:Y10] | AJ781300 | TYLCCNB-CN[CN:Yn10:Tom] |
